# Extracellular matrix stiffness and tumor-associated macrophage polarization: new fields affecting immune exclusion

**DOI:** 10.1007/s00262-024-03675-9

**Published:** 2024-05-02

**Authors:** Ke-Xun Yu, Wei-Jie Yuan, Hui-Zhen Wang, Yong-Xiang Li

**Affiliations:** 1 Department of General Surgery, The First Affiliated Hospital of Anhui Medical University, Hefei, 230022 China; 2 Department of Gastrointestinal Surgery, Xiangya Hospital of Central South University, Changsha, China

**Keywords:** Extracellular matrix stiffness, Cancer-associated fibroblasts, Tumor microenvironment, Tumor-associated macrophage, Immune exclusion

## Abstract

In the malignant progression of tumors, there is deposition and cross-linking of collagen, as well as an increase in hyaluronic acid content, which can lead to an increase in extracellular matrix stiffness. Recent research evidence have shown that the extracellular matrix plays an important role in angiogenesis, cell proliferation, migration, immunosuppression, apoptosis, metabolism, and resistance to chemotherapeutic by the alterations toward both secretion and degradation. The clinical importance of tumor-associated macrophage is increasingly recognized, and macrophage polarization plays a central role in a series of tumor immune processes through internal signal cascade, thus regulating tumor progression. Immunotherapy has gradually become a reliable potential treatment strategy for conventional chemotherapy resistance and advanced cancer patients, but the presence of immune exclusion has become a major obstacle to treatment effectiveness, and the reasons for their resistance to these approaches remain uncertain. Currently, there is a lack of exact mechanism on the regulation of extracellular matrix stiffness and tumor-associated macrophage polarization on immune exclusion. An in-depth understanding of the relationship between extracellular matrix stiffness, tumor-associated macrophage polarization, and immune exclusion will help reveal new therapeutic targets and guide the development of clinical treatment methods for advanced cancer patients. This review summarized the different pathways and potential molecular mechanisms of extracellular matrix stiffness and tumor-associated macrophage polarization involved in immune exclusion and provided available strategies to address immune exclusion.

## Background

The tumor microenvironment (TME) is a heterogeneous environment composed not only of cancer cells, but also of different types of cells and non-cellular components, including tumor-associated macrophages (TAMs), tumor-infiltrating lymphocytes (TILs), cancer-associated fibroblasts (CAFs), blood vessels, and extracellular matrix (ECM) composed of collagen and proteoglycan, which may play a core role in tumor evasion of immune surveillance and resistance to immunotherapy [1, 2]. Among them, ECM is a non-cellular three-dimensional macromolecular network, which components undergo dynamic changes under normal physiological and pathological conditions [3]. ECM is composed of a large number of proteins, including collagens, elastin, fibronectin, laminins, and several other glycoproteins, which regulate diverse cellular functions, such as growth, migration, and differentiation, and are vital for maintaining normal homeostasis [4]. On the other hand, the components of ECM also include proteoglycans (PGs) and glycosaminoglycans (GAGs), and the molecular diversity based on PGs and GAGs leads to the functional diversity of ECM [5]. Due to the ability of PGs and GAGs to bind and activate transforming growth factor β, ECM is induced to participate in inflammatory responses [6]. In addition, PGs and GAGs, as pro-angiogenic and anti-angiogenic molecules, play crucial roles in cell proliferation, migration, and adhesion processes [7]. ECM also plays a crucial role in signal transmission and interaction among cells within the organization [8]. The alteration of density and composition can lead to increased deposition of ECM and changes in tissue structure [9, 10]. At the same time, the deposition of collagen and hyaluronic acid content will increase the stiffness of ECM and transmit physical change signals from ECM to the intracellular matrix; this leads to cancer-associated fibrosis and possibly to a physical barrier to T-cell penetration [11, 12]. Although ECM is strictly controlled during organ homeostasis and cellular communication, in diseases such as cancer, ECM is often deregulated and becomes dysregulated. ECM abnormalities directly promote cell transformation and metastasis, affecting the progression of cancer. Furthermore, ECM abnormalities can also relieve the behavioral regulation of stromal cells, promote tumor-related angiogenesis and inflammation, and further accelerate cancer progression [13]. Therefore, the changes in the stiffness of tumor extracellular matrix not only participate in the occurrence of tumors, but also play a crucial role in the process of tumor metastasis and has the potential to become a new target for cancer treatment.

For a long time, it has been believed that cancer can promote angiogenesis through various pathological processes, accelerate fibroblast proliferation, and increase the expression of growth factors recruited by inflammatory cells to alter ECM [14]. As one of the important components of ECM, CAFs were activated and recruited by various chemokines, playing a crucial role in immune exclusion and tumor metastasis [15]. CAFs have the ability to promote epithelial mesenchymal transition (EMT) of primary tumors by producing in excess an aberrant ECM and altering their mechanical stiffness, providing favorable conditions for distant organ colonization of tumor cells [16]. On the other hand, CAFs affect the progression of potential tumor cells and promote angiogenesis necessary for maintaining epithelial transformation by expressing ECM proteins and growth factors [17]. TAMs are another one of the most crucial components of TME, accounting for over 50% of the immune cells that infiltrate the TME [18]. TAMs are also the main immune cell population that affects tumor development, and it has high plasticity. During tumor progression, TAM can polarize into two main phenotypes: the anti-tumor M1 (TAM1) and the pro-tumor M2 (TAM2). Previous studies have shown that Th2 cytokine IL-4 can induce M2 form macrophage activation, inducing the expression of a range of different surface receptors and effector molecules [19]. In addition, M1 and M2 polarized macrophages are the extremes of continuous activation states in the adaptive response universe. M2 like has been used to refer to phenotypes that have a general relationship with IL-4 activated macrophages [20]. On the other hand, long-term and stable epigenetic markers help define the identity of macrophages and help establish epigenetic memory. The epigenome of macrophages undergoes remodeling under acute and polarized stimuli. This remodeling involves changes in chromatin modifying enzyme expression during macrophage polarization, thereby affecting transcriptional output [21, 22]. The polarization of TAMs from TAM2 to TAM1 phenotype can not only relieve the immunosuppressive constraint and induce cytotoxic T cell immunity, but also improve the sensitivity of tumor cells to chemotherapy, bringing new dawn for cancer treatment. Relevant evidence suggests that CAFs play a role in coordinating the recruitment, survival, and reprogramming processes of TAMs [23]. In a recent study, Mayer et al. revealed a hierarchical network structure of cell interactions in breast TME using single-cell RNA sequencing (scRNA seq) data, with CAF located at the top of the stratification. In order to further explore the dynamics of network regulation in the study, the author used network motif analysis and identified a repetitive dual cell circuit motif. The strongest example of this circuit is CAFs with autocrine circuits and paracrine signals to TAMs, which have weaker paracrine and autocrine signals. In this stable state of the circuit, both cell types will proliferate and die, maintaining a fixed proportion. In the extracorporeal loop, the author identified the potential mediator RARES2 and its receptor CMKLR1 of CAF-TAM interaction, and proved that this mediator enhanced the migration of macrophages in vitro, and was associated with the proto-cancerous TAM phenotype and high tumor grade of breast cancer patients. In addition, the authors identified the chemokine RARRES2 as a paracrine signal sent from fibroblasts to macrophages and from CAFs to TAMs, inducing upregulation of CMKLR1 and macrophage migration, based on cross-transcriptional patterns found in tumor and isolated co-culture systems. Among them, in breast cancer patients, RARES2 is only specifically expressed in CAFs, and it is not expressed in normal fibroblasts or any other cell type in breast TME. This study highlights the existence of CAFs and TAM grading network, in which CAFs play a central role through TAM secretion factors [24]. A more refined understanding of the phenotype and functional procedures of TAMs and CAFs will help improve immune rejection in cancer immunotherapy.

In the past decade, rapid progress has been made in the development of immune checkpoint inhibitors (ICIs), such as programmed cell death protein 1 (PD-1)/programmed cell death-ligand 1 (PD-L1) inhibitors and cytotoxic T-lymphocyte-associated protein 4 (CTLA4) inhibitors, have become a revolutionary cancer treatment method, providing long-term efficacy and survival benefits for a large number of cancer patients [25]. ICIs can block the interaction between ligands and immunosuppressive receptors, thereby overcoming the suppression of immune cells and reactivating cytotoxic immune responses [26]. In addition, with the emergence of new immunotherapy strategies such as chimeric antigen receptor T cells (CAR-Ts) and CAR macrophages (CAR-Ms), the field of immunotherapy has recently entered a new era [27]. However, the adverse clinical reactions to treatment highlight the necessity of understanding and avoiding immunotherapy resistance. Specifically, one of the therapeutic resistance mechanisms of ICIs is the inability of lymphocytes to penetrate the tumor, also known as immune exclusion. The occurrence of this phenomenon may be due to ECM restricting lymphocytes from entering the tumor nest [28]. Research has shown that TME may be involved in tumor treatment resistance by limiting drug acquisition. In fact, due to the involvement of cells such as CAF, a large amount of hard ECM can be secreted. The rigid ECM can promote immune exclusion by limiting the reduction of anti-tumor T cells, becoming a major obstacle to the clinical application of immunotherapy [29]. Furthermore, TAMs can penetrate into TME and express various immune checkpoint molecules, thereby affecting the immune function and the therapeutic effect of ICIs [30]. Up to date, there is insufficient knowledge about the interplay between the ECM and TAMs polarization. In this review, we summarized the changes in extracellular matrix stiffness and the different mechanisms by which the polarization direction of TAMs affects immune exclusion, as well as potential therapies to overcome immune exclusion.

## Potential mechanisms for inducing immune exclusion

### Induction and possible mechanisms of immune exclusion

Immunotherapy mainly exerts anti-tumor effects by blocking the immunosuppressive pathway and promoting adoptive T cell therapy, and is gradually becoming the main complementary treatment method for cancer patients [31]. However, the presence of immune exclusion reduces the drug sensitivity of patients and prevents the further application of immunotherapy in clinical practice. Recent evidence has revealed two potential mechanisms of tumor immune exclusion, namely T cell exclusion within the tumor nest and T cell dysfunction [32]. Generally, higher T cell infiltration into tumors predicts better drug sensitivity and survival for malignant tumor patients. However, only a portion of patients showed clinical reactions to these interventions, and the presence of immune exclusion resulted in a decrease in the response rate of some patients [33, 34]. Immune exclusion means that tumors prevent immune cells from penetrating into the TME, which gradually becomes the key mechanism of drug resistance in immunotherapy [35]. One of the inducing factors of immune exclusion is the existence of physical barriers, which refer to a type of barrier where T cells do not come into direct contact with cancer cells due to mechanical separation and result in the inability to activate immune effector gene features [36]. Increasing studies have shown that CAFs serve as physical barriers and sources of immunosuppressive molecules, contributing to T cell exclusion. The ECM reshaped by CAFs becomes an important barrier that hinders T cells from infiltrating tumor nests. In addition, multiple chemokines were associated with the recruitment of T cells into tumor nests [37], and the expression of appropriate chemokines in the TME was critical for effector T cell trafficking into tumor sites [38]. Accumulating evidence has confirmed that multiple chemokines can be expressed in T cell inflammatory tumors and induce CD8 + effector T cell infiltration within the tumor nest, while non-T cell inflammatory tumors lack the expression of these chemokines [39, 40]. As one of the components of TME, NK cells also play an important role in immune exclusion. Low infiltrating or exclusion of NK cells are often associated with high expression of immunosuppressive genes or immune checkpoint genes, and the resulting tumor immune escape is also considered a major cause of cancer metastasis or progression [41]. In immune exclusion tumors, there may be a gradient of chemokines from the periphery to the center, and the presence of this concentration gradient may limit the infiltration of T cells [42], thereby affecting the efficiency of the immune response.

### Multiple molecular signaling pathways regulate tumor immune exclusion and reduce T cell infiltration in tumor tissue

Next, we explored the molecular signaling pathways that may induce immune exclusion. It has been reported that ECM proteins can regulate the components of Wnt pathway during development and disease, and their imbalance will lead to changes in Wnt signal. Fibronectin is a major ECM protein that regulates β-catenin proteins that are used to regulate classical Wnt information transduction. In addition, integrin, as a fibronectin receptor, also regulates Wnt activity, especially during embryogenesis. On the other hand, Wnt/β-catenin signaling regulates the expression of genes encoding ECM proteins. These pieces of evidence indicate that Wnt/ β-catenin signaling pathway and the secretion and composition of cellular ECM protein components are two closely related processes, and this cross talk change may be closely related to the occurrence of immune exclusion. Activation of β-catenin is considered a key factor in the development of resistance in ICI therapy, and the activation of β-catenin is involved in the formation of immune resistant tumor nest in the TME. Galarreta et al. reported that in a hepatocellular carcinoma (HCC) model the activation of β-catenin pathway can inhibit the recruitment of dendritic cells in TME, thereby inhibiting T cell activation and reducing the infiltration of CD8 + T cells. At this point, the HCC model activated by β-catenin exhibited poor immune cell infiltration and there was immune exclusion in the treatment of PD-1 antibody [43]. An immunohistochemical result in melanoma tissue samples also confirmed that the high expression of β-catenin was mainly found in tumors lacking CD8 + T cells, indicating that there was a significant negative correlation between the activation of β-catenin pathway and a T cell-inflamed tumor microenvironment [44]. One hypothesis was that β-catenin proteins can directly mediate anti-tumor immune responses to achieve immune exclusion. Tumors achieve immune exclusion by eliminating tumor cells that express immunogenic antigens and then upregulating the immunosuppressive pathway that suppresses the function of peripheral affinity residual T cells. In this hypothetical model, the Wnt/β-catenin pathway within tumor cells was activated during immune exclusion, thereby regulating the secretion of related ECM proteins and further altering matrix stiffness. This leads to a decrease in immune cell infiltration in TME, inducing immune exclusion in tumors [45]. Patients who develop secondary resistance to immunotherapy may also be activated β-catenin pathway [46], and the further study of this mechanism may realize targeted therapy for patients with immune exclusion. Another prospective carcinogenic pathway that has a potential impact on tumor immune exclusion was PI3K/AKT signaling pathway. Relevant studies have shown that the activated PI3K signal causes the accumulation of TAMs to increase and then induces the immunosuppressive microenvironment by activating PIK3CA mutations or inducing the loss of PTEN function mutations [47]. Meanwhile, in a study of triple negative breast cancer, it was found that the expression of PTEN was related to the loss of T cells in the TME, which promoted immune exclusion. Therefore, the impact of active PI3K signaling on immune exclusion may vary depending on the tumor and cellular environment [48]. In another study, Ihara et al. constructed epithelium-specific knockout mice model of STAT3, and lung cancer cells showed increased anti-tumor inflammation and natural killer (NK) cell immunity in the absence of STAT3 signaling pathway. This is related to the increased expression of chemokines CCL5 and CXCL10 and the increased accumulation and function of T cells in the TME. The conclusion based on the experimental results was that STAT3 silenced non-small cell lung cancer (NSCLC) cells exhibit enhanced pro-inflammatory chemokine production, reduced MHCI antigen expression, and increased sensitivity to NK cell-mediated cytotoxicity [49]. In addition, the supernatant from STAT3 silenced NSCLC cells promotes the migration of monocyte, which may represent another feasible mechanism to reduce the recruitment of immune cells to tumor sites. In the case of carcinogen-induced tumorigenesis, STAT3 has an inhibitory effect on tumor NK cell immunity and induces subsequent immune exclusion [49]. The above experimental evidence shows that molecular cross-linking of multiple signaling pathways not only promotes the progression of tumor malignant phenotype, but also induces immune exclusion and changes in cell infiltration status in TME. The changes in extracellular matrix stiffness and the adjustment of polarization direction of TAMs involve the activation or imbalance of multiple signaling pathways, which may regulate the infiltration of T cells and the occurrence of immune exclusion in the immune microenvironment.

## Effect of ECM stiffness on tumor progression and immune exclusion

### ECM stiffness and the interaction with the tumor progression

In the past decade, immunotherapy has fundamentally changed the treatment methods of cancer patients and improved their survival rates. However, one of the main obstacles to improving the effectiveness of this treatment method is the increase in the stiffness of tumor ECM, which limits the infiltration of immune regulatory drugs and T lymphocytes in tumor tissue, thereby hindering direct contact between drugs and tumor cells [50]. The regulation of ECM stiffness can affect immune exclusion and the rate of malignant progression of tumors. Research evidence suggests that the ECM stiffness imbalance during cancer progression mainly occurs in stromal cells, including changes in the distribution and content of CAFs and immune cell populations [51]. It is worth noting that the interaction between ECM stiffness and tumor cells is bidirectional: On the one hand, ECM stiffness determines the differentiation, and migration direction of cells, and regulates the interaction between tumor cells. On the other hand, cells actively reshape the composition of ECM and change its stiffness, which is beneficial for tumor progression [52, 53]. In the progression of different types of cancer, the changes in ECM mainly focus on the increase in matrix stiffness and the degradation of corresponding matrices. In fact, a large amounts of CAFs in tumor tissue can secrete abnormally stiff ECM, thereby altering ECM stiffness and promoting immune exclusion and subsequent immune resistance by limiting the aggregation of anti-tumor T cells within the tumor nest [54]. In recent research, Brauchle et al. found that the collagen-rich area in the ECM in colorectal cancer tissue is significantly stiffer than the collagen rich layer under the mucosa of the control tissue. Furthermore, in the collagen network of colon cancer tissue, the arrangement of collagen fibers increases, and the level of glycosaminoglycan (GAG) immune response increases, which may create resistance to immunotherapy and promote the formation of immune exclusion microenvironment [55]. Another study in breast cancer (BC) found that lysine oxidase (LOX), as an enzyme that catalyzes the cross-linking of collagen in extracellular matrix, can increase the matrix stiffness of BC by increasing the cross-linking of collagen. With the increase in the matrix stiffness, local cancer cells will adhere and aggregate, and upregulate GFR-dependent PI3K signal [56], ultimately promoting the progress of BC.

### CAFs alter ECM stiffness and affect immune cell efficacy

As the main characteristic of ECM stroma, CAFs are the chief cell populations responsible for reshaping the stiffness of tumor ECM, promoting tumor growth, regulating disease progression, and influencing treatment response. Significantly, ECM proteins were mainly produced by CAFs, while the main structural proteins of basement membrane, including collagen VI, laminin, and heparan sulfate proteoglycan, were expressed by basal epithelial cells, myoepithelial cell, and CAFs in a tissue-specific manner [57], endowing the tissue epithelium with polarity and specific functions. CAFs are the main type of stromal cells within TME, capable of secreting various cytokines, chemokines, and soluble factors, playing a crucial role in modifying TME and controlling drug entry into tumors [58]. Research has shown that the tumor environment contains a large amount of ECM and CAFs, leading to the production of stiff connective tissue proliferative stromal tumor nests [59]. It is worth noting that the deposition of ECM components in the extracellular space provides favorable conditions for tumor cell migration. Once CAFs establish a microenvironment that promotes connective tissue proliferation, tumor epithelial cells can participate in matrix deposition and subsequent distant metastasis of cancer cells during disease progression [60]. Against the backdrop of the entire continuous changes in TME, the dynamic changes in ECM stiffness are core features that contribute to the physical, biochemical, and cellular characterization of tumors. At this time, the composition and structure of ECM coevolve with tumor progression [61]. In addition, CAFs interact with almost all cells in the tumor microenvironment. Recent studies have found that CAFs promote M2 cell polarization by releasing fibroblast growth factor, and polarized M2 cells express a large amount of TGF-β, promoting fibroblasts to become cancer-related fibroblasts and form a positive feedback loop [62]. The non-tumor cell populations contained in TME, such as CAFs and macrophages, also affect tumor immune escape and alter the response rate and survival time of immunotherapy [63, 64]. On the other hand, CAFs are beneficial for the exclusion and depletion of T cells, promoting the recruitment of bone marrow-derived inhibitory cells, including monocytes and the resulting macrophage population and inducing pre-tumor phenotype changes in macrophages and neutrophils [65]. Tumors lacking cytotoxic T lymphocyte infiltration are termed immune-excluded tumor, which are particularly resistant to various types of treatments [66]. On the other hand, it was found in mouse tumor models that the tumor core that experiences immune exclusion lacks infiltration of CD8 + T cells, but there is a small accumulation of CD8 + T at the edge of the tumor, which is insufficient to induce an effective immune response that may be caused by T cell depletion, and even induce resistance to treatment [67]. The mechanism by which tumor nests restrict T lymphocyte infiltration may be related to the production of dense collagen and hyaluronic acid by CAFs, as well as the secretion of inhibitory cytokines such as TGF-β and CXCL12, and T cells are confined to the matrix to prevent their accumulation near cancer cells, thereby inhibiting anti-tumor immunity [68]. Secondly, immune exclusion results in insufficient blood supply to the blood vessels within the tumor nest and almost no expression of lymphocyte recruitment signals, such as CCL19, CCL21, CXCL9, and CXCL10, reducing lymphocyte infiltration and migration [69]. Galon et al. observed in their study that the infiltration of CD8 + T cells in tumor nests and the presence of adjacent cancer cells predicted an improvement in survival rate in colorectal cancer (CRC) patients, and the predictive accuracy was higher than the classic TNM staging system [70]. Additionally, within the TME, dynamic interactions between different cell types and CAFs regulate and support tumor progression by altering ECM stiffness. In particular, these interactions limit the response to anti-tumor therapy and provide a protective barrier against immune monitoring and the spread of anti-tumor factors [71], creating favorable conditions for immune exclusion.

### Reshaping ECM stiffness to optimize tumor immune exclusion

ECM stiffness has been proved to help induce immune exclusion, which can predict poor prognosis in various malignant tumor patients [72]. Therefore, targeted strategies to reduce ECM stiffness have considerable potential in tumor treatment. High collagen deposition is one of the main characteristics of desmoplastic TME, making tumors stiffer than normal tissue [56]. The various sources of TME cells and different stimuli lead to the production of different subpopulations of fibroblasts, mainly α‐smooth muscle actin (α-SMA)-positive ECM-derived myofibroblastic CAF (ECM-myCAFs) [73]. ECM-myCAFs are typically located near tumor tissue and exhibit a matrix secretory phenotype with high survival rates of motor neuron 1 (SMN1, SMA) expression. They play a regulatory role in the expression of immune regulatory secretions such as interleukin-6 (IL-6) and chemokine (C-X-C motif) ligand 12 (CXCL12) [74]. Due to the heterogeneity of CAFs and the multiple roles of ECM-myCAFs in regulating immune suppression and altering tumor immune continuity, co-targeted CAFs immunotherapy has become a popular choice for cancer treatment [75]. Tumor cells can reshape and organize the secretion form of these fibers and collagen by producing highly arranged collagen fiber regions, which provides a viscous trajectory for tumor cell migration, reduces the energy required for migration, and promotes tumor cell infiltration [76]. The changes in collagen expression patterns have been confirmed to be associated with poor prognosis in various tumors, and the reduction in type I collagen synthesis mediated by CAF shows an inhibitory effect on tumor growth [77, 78]. Therefore, diagnostic tools based on ECM component and stiffness analysis can help to achieve patient risk stratification, identify chemotherapy resistance and immune exclusion subgroups, improve the overall treatment effect of cancer patients, and extend the survival time [79]. Furthermore, the remodeling of tumor ECM is also considered a crucial factor in controlling immune cell infiltration, differentiation, activation, and polarization of TAMs, playing a core role in inducing immune exclusion during tumor treatment [80]. The biophysical methods of inducing ECM remodeling can also promote immune monitoring and improve the success rate of immunotherapy, playing an inhibitory role in tumor progression. In a preclinical model of cholangiocarcinoma (CCA), the time-limited light (photothermal therapy) or electromagnetic (magnetothermal therapy) activation of intratumorally implanted biodegradable gold–iron oxide nanoflowers (GIONFs) nanoparticles can significantly reduce ECM stiffness within the tumor, leading to depletion of CAF and significant ECM remodeling, which has a certain inhibitory effect on the growth and migration of cancer cells [81]. In a recent study on pancreatic ductal adenocarcinoma (PDAC), Gong et al. found that the expression of stemness-related genes (SOX2, OCT4 and NANOG) in the stiff group (10% GelMA) showed a significant increase and spheroid formation compared with the soft group (5% GelMA) when the methacrylate gelatin (GelMA) hydrogel with adjustable stiffness was used to incubate pancreatic cancer cells. In addition, it was also observed that the stiffness of ECM regulated the autophagy process of PDAC cells, which plays an important role in promoting the stemness of PDAC cells, leading to a poorer prognosis for patients. The use of rapamycin and chloroquine can block autophagy while inhibiting cell stem development induced by ECM stiffness changes in PDAC, providing a potential therapeutic strategy for this invasive cancer [82]. A study in BC shows that the transformation from non-malignant tissue to invasive ductal carcinoma corresponds to significant collagen deposition and fibrotic arrangement, resulting in ECM stiffen of tumor cells. Subsequently, these changes enhanced the activation of mechanical sensitive signaling pathways related to focal adhesion and growth factor receptors, while significantly enhancing the infiltration of “activated” tumor promoting macrophages. On the contrary, the number of infiltrating T lymphocytes in tumor tissue decreased, and the ECM stiffness induced immune exclusion in the tumor nest of BC. Further studies have concluded that the infiltrating immune cells in BC secrete abundant TGF-β to stimulate tumor and matrix TGF-β signals induce the production of collagen and collagen cross-linking enzyme lysine oxidase (LOX), which provides a possible mechanism for the fibrosis and ECM stiffness. In the follow-up research, we need to continue to explore how the changes in the invasion of the inherent matrix and immune cells affect the progress and treatment of tumors, and combine the tumor behavior with the mechanical changes of ECM to reduce the probability of immune exclusion in the treatment of BC [83]. To sum up, it is obvious that the dynamic changes in ECM stiffness were involved in regulating immune exclusion and tumor malignant progression. Therefore, targeted CAFs can regulate the secretion of fibrin and reshape the ECM collagen network structure and stiffness, which may bring dawn to cancer treatment.

## Polarization of TAMs involved in immune exclusion

### Polarization of TAMs and its clinical significance

The importance of TAMs in tumor development and immunotherapy has been increasingly recognized. It has been confirmed that TAMs not only directly provide structural support for the development of cancer, but also participate in exclusion of immunotherapy by secreting signaling molecule and extracellular vesicles (EV) [84]. TAM has two polarization states, among which TAM1 is characterized by the secretion of pro-inflammatory cytokines such as TNF-α, interleukin-1 (IL-1), IL-6, IL-8, and IL-12, and promote anti-tumor immune response [85]. And TAM2 is an important component of immunosuppressive TME and secretes TGF-β inducing regulatory T cells (Treg) and inhibiting CD8 + T cell response to reduce cancer cell death [86]. Inhibiting TAM2 while enhancing the polarization of TAM1 can not only activate cytotoxic T cell, but also directly induce TAM1 to secrete anti-tumor cytokines, which can regulate the anti-tumor immune response [87]. Growing evidence has shown that TAMs not only secretes cytokines and survival factors, but also guides the interaction between cancer cells and macrophages through ECM deposition and remodeling, promoting the resistance of cancer cells to chemotherapy and radiation therapy [88, 89]. And the infiltration of TAMs has been proved to be involved in tumor angiogenesis, invasiveness, and immunosuppressive processes. For example, removing TAMs by inhibiting CSF1 can significantly reduce the angiogenesis potential of breast cancer [90]. On the contrary, when CSF1 levels were rescued, TAMs depletion was blocked and the potential for angiogenesis was enhanced [91]. In addition, TAMs can promote the generation of lymphangiogenesis in tumors. Hwang et al. reported that the high expression of VEGF-C in M2 like TAMs promoted lymphangiogenesis [92]. In the process of cancer metastasis, TAMs mainly promote the invasion and migration of tumor cells by secreting matrix metalloproteinases, serine proteases, and cathepsins, which modify the cell–cell junctions and destroy the basement membrane to promote the distant metastasis of carcinoma in situ cells [93]. A recent study in pancreatic cancer shows that the increased invasion of TAM2s can promote the EMT process of pancreatic cancer by activating TLR4/IL-10 signaling pathway in cancer cells, reducing E-cadherin and increasing the expression of Vimentin [94]. More and more evidence also suggests that TAMs are associated with cancer chemotherapy and radiation resistance. The inhibition of TAM2s directional polarization can weaken the resistance to chemotherapy and radiotherapy in vivo and in vitro [95], and is closely related to the improvement of survival prognosis in tumor patients. TAMs and their polarization directions regulate various pathological progression processes of tumors. Further research on TAMs can lead to the development of targeted treatment strategies for cancer development and the reduction of immune exclusion.

### TAMs regulate immune exclusion and influence ICIs effects

At present, immunosuppression has become increasingly common in the treatment of cancer, and the infiltration of TAMs in cancer tissue is one of the main reasons for inducing immunotherapy resistance [96]. TAMs promote immune suppression of TME by inhibiting T cell activity and inducing the expression of immune checkpoint ligands [97], which is associated with low immune therapy response and reduced overall survival rate (OS) in tumor patients [98]. Furthermore, with the increase in the ECM stiffness, the accumulation of immunosuppressive factors in TME can attract Tregs and polarizes macrophages to an M2 pro-tumoral phenotype [99]. Specifically, TAM2 is a subtype of macrophages that promotes tumor progression and can be identified through various surface markers, including CD163, CD206, Fizz1, and Arg1 [100]. As positive feedback, the enrichment of Tregs and M2 macrophages promotes the production of immunosuppressive microenvironment and the exclusion of immune drug infiltration [101]. Therefore, targeting immune checkpoints in TAMs can enhance the effectiveness of immune therapy. This remodeling of tumor extracellular matrix by regulating the direction of TAM polarization has become a new therapeutic strategy, which is expected to improve the prognosis of tumor patients [102]. Given the crucial role of TAMs in the expression of immune checkpoint molecules and their subsequent regulation of immune exclusion, we summarized the functions and regulatory pathways of immune checkpoint expression in TAMs. Firstly, high expression of PD-1 in TAMs regulates macrophage polarization and phagocytosis. In macrophages overexpressing PD-1, TAM1-related markers such as iNOS were downregulated, while TAM2-related marker Arg-1 was upregulated. This may be due to the upregulation of STAT6 pathway by PD-L1 expressing T cells to polarize macrophage polarization into M2 phenotype [103]. Another study in the melanoma mouse model found that the expression of PD-1 on TAMs showed significant circadian rhythm fluctuations and was regulated by Dec2. Mechanically, Dec2 periodically suppressed NF-κB-induced transactivation of the Pdcd1 gene, resulting in diurnal expression of PD-1 in TAMs. Furthermore, Dec2 inhibits the expression of macrophage PD-1 by constricting p65 nuclear translocation, thereby affecting the anti-tumor effect of macrophages [104]. Then, the difference from PD-1 is that macrophages expressing PD-L1 exhibit double-sided characteristics, and their impact on the immune microenvironment is mainly manifested as immunosuppressive function [105]. After treatment with anti-PD-1/PD-L1 antibodies, TAMs showed enhanced phagocytic activity, thereby restoring their anti-tumor function. A study in glioblastoma multiforme (GBM) found that TAMs with high PD-L1 expression were significantly related to M2 polarization, and secreted typical chemokines, TGF-β And IL-10. Experimental data confirm that PD-L1-mediated immunosuppression may be attributed to the infiltration of TAMs and M2 polarization, and is closely related to the poor prognosis of GBM patients [106]. Eventually, CTLA4 is a transmembrane protein expressed in activated CD4 + and CD8 + T cells, and the checkpoint CTLA-4 is expressed by T cells, including high and constitutive expression by Tregs [107]. Although the inhibitory signal of CTLA4 negatively regulates T cell initiation and may induce immune rejection within the tumor nest, it subsequently induces PD-1-mediated T cell activation and proliferation, resulting in a killing effect on tumor cells [108]. In the course of treatment, it was found that targeting PD-1 led to the expansion and recruitment of anti-tumor T cells, while anti-CTLA4 therapy would produce new T cell clones, indicating that CTLA4 and PD-1 might simultaneously target the synergistic anti-tumor effect, and the anti-tumor effect of their combination therapy was better than that of single therapy [109]. Kim et al. found a high degree of spatial heterogeneity in the tumor immune microenvironment (TIME) of high-grade glioblastoma (HGG), where depleted CD8 + T cells and immunosuppressive cells, including Tregs and TAM2s, are more abundant in the core region than in the peripheral region. In vitro cultivation of infiltrating immune cells in the peripheral region under hypoxic conditions leads to an increase in end-depleted CD8 + T cells, CTLA-4 + TREG cells, and TAM2s, which is significantly correlated with a decrease in progression free survival in HGG patients and directly induces immune exclusion, leading to a poorer therapeutic effect of CTLA4-related inhibitors [110]. In addition to the significant differential distribution of immune cells in the core and peripheral regions of the tumor, Kim et al. also found an enhanced hypoxic feature in the core region. Meanwhile, previous studies have shown that hypoxia induces changes in ECM composition and stiffness (manifested as changes in fibronectin content and multiple ECM secreted proteins), followed by alterations in the expression of integrins that interact with ECM, promoting cancer cell migration and providing a fundamental condition for subsequent immune exclusion [111]. The in-depth development of treatment methods targeting TAMs polarization and macrophage infiltration in the future is expected to reduce immune exclusion in tumor immunotherapy, enhance drug sensitivity, and improve the long-term survival rate of cancer patients.

## The tri-directional interactions of ECM stiffness, TAMs polarization and immune exclusion in terms of tumor progression

**Fig. 1:**
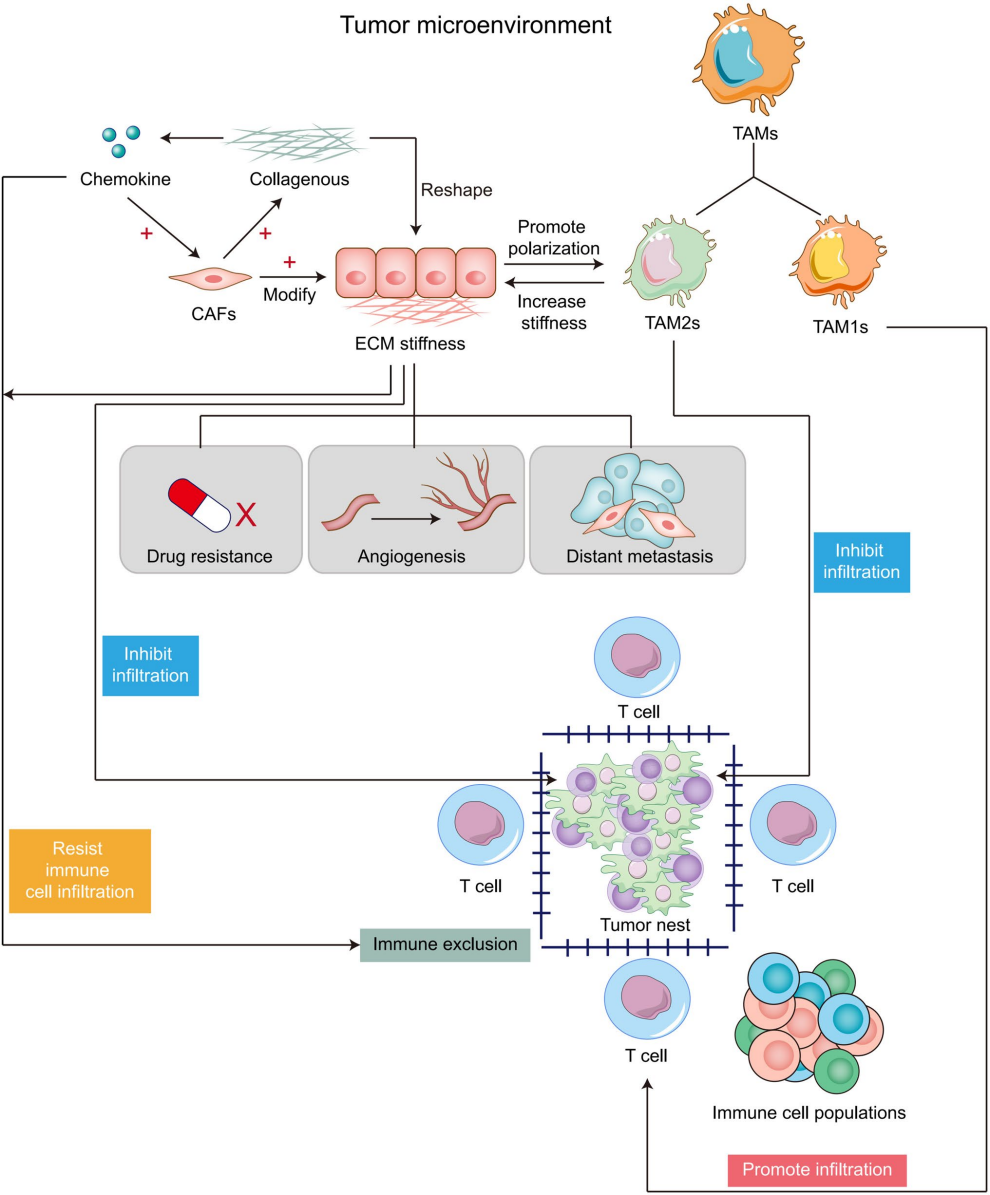
Tri-directional interactions of ECM stiffness, TAMs polarization, and immune exclusion in terms of tumor progression

ECM stiffness and polarization of TAMs play a key regulatory role in tumor immune exclusion, and changes in ECM stiffness also affect the polarization direction of TAMs and participate in various pathological progression processes of tumors (Fig. 1), making them potential therapeutic targets. Multiple cell types, including epithelial cells, CAFs, immune cells, endothelial cells, TAMs, etc., synthesize and secrete matrix macromolecules under the control of multiple signals, thereby participating in the formation of ECM in various tumors and regulating ECM stiffness [112, 113]. Compared with ECM in normal tissues, the secretion of tumor-related ECM molecules leads to significant differences in TME composition and tissue, and alters the establishment of tumor niches, angiogenesis, and immune response, enhancing tumor resistance and exclusion in immunotherapy [8, 114]. Relevant evidence indicates that during the malignant progression of various tumors, ECM components undergo significant changes, leading to the formation of fibrotic matrix and promoting cross-linking between collagen fibers and ECM components, leading to an increase in matrix stiffness [8]. On the other hand, CAFs, TAMs, and tumor cells themselves in the tumor matrix were involved in the deregulation and formation of unorganized ECM that was conducive to tumor development. These abnormalities make ECM molecules and related cells potential targets for targeted drug therapy [115]. A previous study found in a colorectal cancer mouse model that the TAM2 population expressed significantly more PD-1 than the TAM1 population, and the percentage of PD-1 TAMs was positively correlated with tumor size, exhibiting phagocytic inhibition. In addition, the expression of TAM PD-1 increases over time in mouse models and with disease staging in primary human cancers. Further block PD-1/PD-L1 axis in vivo to enhance Phagocytosis in a macrophage-dependent manner, inhibit tumor growth, and prolong the survival period of mouse tumor model [98]. In the triple negative breast cancer model constructed on CD169-DTR mice, TAMs support tumor growth and metastasis. CD169 macrophage depletion alleviated tumor-induced splenomegaly in mice. Further data show that breast cancer cells and CD169 macrophages show two-way interaction, which plays a key role in tumor progression by activating JAK2/STAT3 signaling pathway in macrophages. The impact of CAFs on tumor progression, drug delivery, and efficacy is related to the production of a large amount of stiff ECM [116]. The overflow of ECM components in the extracellular space provides favorable conditions for the metastasis and colonization of tumor cells [117]. Once CAFs establish a microenvironment that promotes connective tissue proliferation, epithelial tumor cells can promote matrix deposition and change ECM stiffness in the later stages of tumor progression, inducing immune exclusion [118]. Another important regulatory factor for ECM composition and matrix stiffness is the expression of cytokine transforming growth factor-β (TGF-β). This molecule is mainly secreted by CAFs, but can also be produced by myeloid cells and tumor cells [119]. Tumor cell-derived TGF-β, platelet-derived growth factor (PDGF) and fibroblast growth factor (FGF) can activate tissue fibroblasts [120]. ECM dysregulation during cancer progression was mainly carried out by stromal cells, including CAFs and TAMs [121]. In fact, fibroblasts are likely to be activated in the early stages of cancer transformation and produce CAFs, which actively participate in tissue remodeling and promote further proliferation of epithelial cells and activation of stromal cells, which in turn exacerbates the development of cancer. In addition, CAF-derived factors can also promote treatment resistance and immune exclusion [122]. TAMs polarization is a process that responds to the expression of different functional programs in microenvironmental signals. There are various polarization states based on functional states that can obtain specific phenotypes during tumor progression [123]. More and more studies have shown that TAMs can enhance or antagonize the anti-tumor effects of cytotoxic chemotherapy, cancer cell targeted antibodies, and immunotherapy agents, depending on the treatment mode and tumor model. TAMs can also drive the tumor repair mechanism after radiotherapy or vascular targeted drug therapy, and regulate the therapeutic response in the direction of macrophage polarization [124]. TAM2 promotes extracellular matrix remodeling by secreting proteolytic molecules or stimulates tumor cell proliferation, migration, and invasion by non-proteolytic proteins, directly promoting tumor occurrence, development, and metastasis [125]. In addition, TAM2 interfere with the anti-tumor function of other immune cells, further inducing the immune exclusion effect of cancer cells [126]. Research has shown that M2 like TAMs are mostly present in the perivascular and hypoxic regions of different mouse and human tumors [127]. At this time, a group of angiogenesis-related TAMs required for tumor angiogenesis, also known as monocyte/macrophages (TEMs) expressing TIE2, showed a serious M2 deflection phenotype, characterized by increased expression of MRC1 and CD163, while the expression levels of MHCII molecules and pro-inflammatory cytokines were relatively low [128]. Meanwhile, the TAMs with a relatively M1 skewed phenotype may appear more frequently in the necrotic regions of early or receding tumors as well as progressing tumors [129]. TAMs can also regulate tumor response to chemotherapy drugs in various ways. The anti-tumor activity of taxane docetaxel in breast cancer involves the depletion of immunosuppressive (M2 like) TAMs in tumor tissue and the concomitant activation or expansion of anti-tumor (M1 like) monocyte/MDSCs. In addition, T cell assay showed that monocyte/MDSCs treated with docetaxel could enhance tumor specificity and cytotoxic T cell response [130]. On the other hand, the sources of CAFs are heterogeneous, and different subtypes of CAFs have been identified in tumors from different sources [131]. It is reported that four different CAF subtypes (CAF-S1 to CAF-S4) have been identified in breast cancer and ovarian cancer [132, 133]. Among them, CAF-S1 enhances the migration of tumor cells and the initiation of EMT through signal cross talk involving both CXCL12 and TGF-β, while CAF-S4, due to its high contractility, is conducive to the invasion and movement of cancer cells [134]. In addition, high levels of CAF-S1 and CAF-S4 subsets have been observed in invasive breast cancer (triple negative and HER2) and metastatic lymph nodes, highlighting their effects in immune exclusion and tumor promotion [132]. CAF-S2 and CAF-S3 account for a relatively small proportion in the subcellular classification of CAF, and may further affect matrix composition and stiffness by regulating the secretion of fibrin, thereby promoting the formation of a tumor suppressive microenvironment [135]. At present, targeted therapy aimed at remodeling TME has shown promising therapeutic results in some cancer types. For example, navitoclax is an inhibitor of Bcl-XL/Bcl-2 protein that can trigger cell death. However, tumor cells can develop resistance to navitoclax by increasing the expression of the anti-apoptotic factor Mcl-1. In relevant experiments, it was observed that CAFs are sensitive to navitoclax. At this time, navitoclax targeting CAFs may achieve therapeutic effects by reducing the proliferative response of connective tissue, ECM deposition [136], and inhibiting tumor growth, which opens up new avenues for tumor treatment.

On the other hand, understanding the signal cascade of mechanical signals transmitted from the plasma membrane to the cytoplasm and then to the nucleus to generate mechanical response transcriptomes has become a research focus. YAP and its homologous TAZ, also known as the Hippo pathway effector, have been identified as key mechanical transducers in the transcriptional program that perceive mechanical stimuli and transmit signals to control cell proliferation, differentiation, and transformation [137]. In addition, activation of the classic Hippo pathway kinase LATS1/2 can phosphorylate YAP/TAZ and promote its cytoplasmic retention and proteolytic degradation. Mechanical cues can directly regulate the activation of YAP/TAZ by releasing the chelating effect of the nuclear ARID1A-SWI/SNF complex on YAP/TAZ [138]. Non-phosphorylated YAP/TAZ shuttle into the nucleus and bind to transcription factors, especially TEAD, to drive target gene expression. The change in ECM stiffness promotes actin polymerization and stress fiber formation. The Hippo kinase cascade is inactivated by actin polymerization and stress fiber formation under high ECM stiffness [139]. On the other hand, the aggregated nuclear actin binds to the ARID1A-SWI/SNF complex under high ECM stiffness, and then releases its chelation with YAP/TAZ. Stiffness activated JNK can phosphorylate LIMD1, which directly binds to LATS1/2 and reduces its kinase activity, thereby activating YAP/TAZ [140]. Furthermore, the Hippo signaling components also regulate the tumor microenvironment through interactions with immune cells such as macrophages. YAP also directs the recruitment of TAMs, which is crucial for immune evasion and tumor development [141]. Similar to YAP, TAZ also promotes the production of inflammatory cytokines and the infiltration of macrophages, playing a regulatory role in tumor progression. A recent study has shown that the expression of YAP and TAZ in macrophages increases in response to pro-inflammatory or repair stimuli. Knockout or conditional gene deletion of YAP/TAZ in macrophages leads to reduced expression of pro-inflammatory genes and increased expression of anti-inflammatory/repair genes. YAP/TAZ promotes pro-inflammatory phenotype by directly regulating the activity of the interleukin-6 (IL6) promoter or through the p38-dependent MAPK pathway. While, during the repair phenotype, YAP/TAZ inhibits the expression of Arg1 by binding to its promoter and recruiting the histone deacetylase 3 (HDAC3)-nuclear receptor corepressor 1 (NCoR1) repressor complex [142]. In addition, Shen et al. found that the Hippo pathway is associated with TGF-β signal transduction to coordinate the cellular or functional transformation of adipocytes from energy storage to ECM remodeling in adipose tissue fibrosis. They found that knocking out LATS1/2 in adipocytes can enhance TGF-β stimulate to differentiate into DPP4 progenitor cells and transform into DPP4 myofibroblasts. On the other hand, the inhibition of the Hippo pathway during obesity weakens the characteristics of adipocytes and promotes the remodeling activity of adipocyte ECM. Macrophages recruited by CCL2 produce TGF-β to accelerate fibrosis of adipose tissue. Importantly, inhibiting YAP/TAZ activity significantly alleviates adipose tissue fibrosis and improves metabolic homeostasis in obese mice [143]. Another study showed that disabled homolog 2 mitogen-responsive phosphoprotein (DAB2) is highly expressed in tumor-infiltrating TAMs, and its gene ablation significantly impairs the formation of lung metastasis. TAMs expressing DAB2 are mainly located at the forefront of tumor invasion and participate in integrin recovery, ECM remodeling, and targeted migration in the three-dimensional matrix. Macrophages expressing DAB2 promote invasive transmission of cancer cells through the mechanosensory pathway of transcription factor YAP. Therefore, DAB2 is the center of TAMs promoting malignant phenotype and is crucial for the formation of metastasis [144]. These conclusions suggest that by activating the complex YAP-TAZ, DAB2 in macrophages can be regulated, thereby controlling integrin recovery and ECM remodeling in the three-dimensional tissue matrix, ultimately regulating tumor cell migration and immune exclusion, which may bring new approach for improving immunotherapy resistance.

## Regulating ECM stiffness and polarization direction of TAMs as a remedial strategy for immune exclusion

### Targeted ECM stiffness therapy improves tumor immune exclusion

Although the clinical application of immunotherapy has entered a new stage of cancer treatment, ICIs based immunotherapy still faces challenges. On the one hand, the response rate of immunotherapy alone is relatively low, and there are significant differences in response among different individuals and tumor types [145]. On the other hand, the serious side effects and the lack of enough tumor-infiltrating lymphocytes in the treatment lead to the reduction of drug response rate [146]. To accurately predict the treatment response of tumors to ICIs, it is necessary to understand how tumors escape immune system surveillance, and promote the transformation of immune exclusion phenotype tumors into inflammatory tumors to reduce resistance to ICIs [147–149]. Although there is a high level of CD8 + T cell infiltration in some tumor nests, these cells are dysfunctional and unable to exercise normal immune function. In other tumors, immunosuppressive factors may exclude T cells from infiltrating tumors, allowing tumor cells to evade immune surveillance [46]. As a potential solution, a computational model for tumor immune dysfunction and exclusion (TIDE) can be used to explore the underlying factors of tumor immune rejection mechanisms. TIDE model integrates and simulates the data of 189 human cancer studies, and verifies the accurate genetic characteristics to simulate tumor immune rejection, which can be used as a reliable alternative Biomarker to predict ICIs response [32]. This can serve as a potential model for predicting and potentially addressing immune rejection. Le et al. described the characteristics of immune infiltration in primary CRC through immune scoring, which can refine and expand the proportion of stage IV patients who meet the treatment conditions of immune checkpoint inhibitors [150]. Immune scoring can also identify stage II CRC patients with high-risk clinical pathological features and satisfactory prognosis, which can avoid adjuvant treatment [151]. This once again demonstrates the clinical practicality of immune scoring in avoiding immune exclusion. On the other hand, degradation of tumor ECM fibers can achieve a decrease in matrix stiffness and normalization of TME. Moreover, the use of collagenase in preclinical cancer models can lead to the breakage of collagen fibers arranged around the tumor matrix and enhance T cell migration [152]. In a study of CCA, it was found that the destruction of collagen matrix in ECM was coordinated with the inhibition of collagen fiber cross-linking. The lower ECM stiffness and structural arrangement enhance the infiltration and movement of T cells, increase direct contact between tumors and T cells, and improve the efficacy of anti-PD-1 immunotherapy [153]. Therefore, reducing the stiffness of ECM around cancer cells and decreasing the cross-linking of collagen fibers in tumor tissue can serve as potential therapeutic options for diluting immune exclusion. In a recent study, tumor cell-derived microparticles co-delivering calcipotriol and indocyanine green have been developed to regulate CAFs to improve the efficacy of photothermal therapy (PTT), and provide a potential method for combining PTT to completely eliminate tumors and improve immune exclusion by remodeling TME through CAFs [58]. At this point, calcipotriol and indocyanine green can effectively target tumor tissue and regulate CAFs to reduce tumor ECM production and stiffness, thereby generating strong PTT efficacy, activating CD8 + T cell-mediated anti-tumor immunity [58], and triggering long-term anti-tumor immunological memory to inhibit tumor recurrence and metastasis. Currently, most drug research on tumors is conducted in cellular and animal models, but the biophysical barriers to drug delivery have been overlooked. The changes in ECM stiffness often weaken the delivery efficiency of these drugs in tumor tissue, leading to poor therapeutic effects. In a PDAC study, it was found that the angiotensin II receptor blocker losartan has a satisfactory application in improving the efficacy of gemcitabine. Losartan has been shown to have the characteristic of consuming ECM. It increases the hydraulic conductivity of the culture gap and makes the culture matrix have more gaps, while the collagen content decreases. This improves the effectiveness of gemcitabine and also demonstrates the importance of establishing a tumor biophysical barrier model for successful evaluation of new drugs and delivery methods [154]. In addition, the chemotherapy resistance mechanism mediated by ECM is mainly based on the activation of integrin signaling, which leads to overexpression of pro-survival and anti-apoptotic proteins, cell cycle arrest, regulation of drug efflux, and phenotype transition of cancer cells (such as EMT or cancer stemness), which is called cell adhesion-mediated drug resistance (CAM-DR) [155]. Targeted activation of CAFs is another strategy to alleviate ECM remodeling mediated by CAFs and reduce tumor stiffness. For example, vismodegib (or saridegib) and pirfenidone, which target Hedgehog and TGF-β pathway activation in CAFs, respectively, has been shown to enhance the efficacy of chemotherapy and immunotherapy in breast and pancreatic tumor models in vivo [156]. In another study, the author utilized the CAF reprogramming ability of tranilast to develop micelles loaded with tranilast. Compared with free drugs, a 100 fold reduction in the dose of tranilast induced better reprogramming in microcytes, due to increased drug accumulation and uptake of CAFs within the tumor. Combination of tranilast micelles and epirubicin micelles or Doxil with immunotherapy increases T-cell infiltration, resulting in cures and immunological memory in mice bearing immunotherapy-resistant BC. In addition, shear wave elastography (SWE) can monitor the decrease in tumor stiffness caused by tranilast micelles and predict the response to nanoimmunotherapy. Micelle encapsulation is a promising strategy for TME reprogramming, providing a new model for cancer treatment [157]. In addition, the combination of tranilast and docetaxel can significantly reduce ECM components, increase tumor vascular diameter and pericellular coverage. These modifications lead to a significant increase in tumor perfusion and oxygenation, and enhance therapeutic efficacy. Tranilast further normalizes immune TME by restoring T cell infiltration and increasing the proportion of T cells migrating away from immunosuppressive CAFs. The combination of tranilast and docetaxel nanomedicine significantly increased the content of immunostimulant M1 macrophages in tumorigenic tissues and enhanced the efficacy of immune checkpoint blocking antibodies against PD-1/CTLA-4 [158]. Recent studies have also shown that in preclinical tumor models of PDAC and BC, mechanical therapy, such as pirfenidone, losartan, tranilast and dexamethasone, can reduce the stiffness and mechanical force of ECM, increase tumor perfusion and drug delivery, thus improving the drug sensitivity of tumor patients. By using two orthotopic mammary tumor models, Polydorou et al. demonstrated that pirfenidone can reduce collagen and hyaluronan levels and, significantly increase vascular function and perfusion levels, thereby enhancing the anti-tumor efficacy of doxorubicin. The reduction of ECM components and normalization of TME are achieved through TGF-β signal pathway inhibition mediated, originating from downregulation of TGF-β1, COL1A1, COL3A1, HAS2, and HAS3 expression levels. The results of this study demonstrate that the use of pirfenidone can be a promising strategy to enhance drug delivery to solid tumors by normalizing the TME, thereby enhancing the therapeutic effect of anti-tumor drugs [159]. In another study, it was found that dexamethasone normalized the stiffness of blood vessels and ECM, thereby reducing interstitial fluid pressure, tissue stiffness, and solid stress. At this point, the permeability of dextran, which represents the nanocarrier, increases. The mechanism model of fluid and macromolecular transport in tumors constructed indicates that dexamethasone increases the permeability of nanocarriers by increasing interstitial hydraulic conductivity, without significantly reducing the effective pore size of blood vessel walls. In addition, dexamethasone increased tumor accumulation in ~ 30 nm polymeric micelles containing cisplatin (CDDP/m) and its efficacy in mouse models against primary BC and spontaneous BC lung metastasis. These results indicate that pre-treatment with dexamethasone before nanocarrier administration can improve the therapeutic effect on primary and metastatic tumors [160]. Currently, drugs used to target ECM stiffness and improve tumor therapeutic effects have shown satisfactory results in preclinical models (Table 1). Further in-depth research in the future can promote the combined use of such drugs with chemotherapy or immune drugs in clinical treatment, effectively improving ECM delivery of chemotherapy drugs and infiltration of immune cells in tumor nests.

**Table 1:** Related drugs or materials involved in regulating ECM stiffness and improving anti-tumor efficacy

Drugs or materials	Combination drugs	Therapeutic target	Mechanisms	References
Collagenase	ICIs	Collagen matrix	Reducing ECM stiffness Increasing T cell infiltration	[152, 153]
Calcipotriol & indocyanine green	Photothermal therapy	CAFs	Remodeling TME Activating CD8 + T cell-mediated anti-tumor immunity	[58]
Losartan	Gemcitabine	Collagen content	Increasing the gap between culture media Consuming ECM and reducing collagen content	[154]
Vismodegib & pirfenidone	Chemotherapy and immunotherapy drugs	Hedgehog and TGF-β pathway activation in CAFs	Relieving ECM remodeling mediate by CAFs and reducing tumor stiffness	[156]
Tranilast	Epirubicin and Doxil	CAFs reprogramming	Increasing drug accumulation and uptake of CAFs	[157]
Pirfenidone	Doxorubicin	Collagen and hyaluronan	Reducing ECM components Increasing blood vessel functionality and perfusion	[159]
Dexamethasone	Cisplatin	Vessels and ECM	Reducing interstitial fluid pressure and tissue stiffness Increasing drug accumulation in tumors	[160]
Tranilast	Docetaxel and ICIs	Immune TME	Increasing the content of immunostimulant M1 macrophages	[158]

### Regulating the polarization direction of TAMs and reducing immune exclusion

The characteristic of TAMs having different effects as the polarization direction changes makes them attractive targets for regulating TME and achieving tumor regression or improving current therapeutic efficacy [161]. Exhaustion of TAM, reprogramming, and inhibition of its recruitment are some proposed targeted therapies [162]. Modulation of TAMs by regulating M1 signaling activation has emerged as a promising and novel immunotherapy strategy. Promoting the conversion of TAMs from M2 to anti-tumor M1 type can alter the mesenchymal characteristics of related cancer cells, and as a potential therapeutic approach, it may play a role in reducing tumor immune exclusion. Macrophage polarization direction regulation has become an independent synergistic factor in cancer progression [163]. At present, potential therapeutic measures mainly focus on three strategies: blocking the recruitment of macrophage precursors, depletion of TAMs and their progenitors, and reprogramming of macrophage function in tumors [164]. Research has shown that cross talk between TAMs and tumor cells can regulate the induction of the pluripotent gene SOX-2 through EGF receptor-mediated STAT3 signaling activation, and plays a crucial role in the expansion of cancer stem cells, chemotherapy resistance, and immune exclusion [165]. In the pancreatic cancer model, blocking the recruitment of TAM by activating STAT3 will also reduce the number of cancer stem cells. These characteristics make TAMs independent targets in TME [166]. Therefore, targeting TAMs may improve the response of chemotherapy resistant solid tumors to conventional chemotherapy. A new therapeutic strategy is chimeric antigen receptor macrophages (CAR-Ms), which can polarize TAMs into M1 anti-tumor subtypes, enhance the phagocytosis of cancer cells [167], and transform TME into an inflammatory cell environment, reflecting the anti-tumor effect in preclinical model validation [168]. Another treatment strategy involves reprogramming TAMs, which can switch between M1 or M2 phenotypes when there are changes in cytokine secretion present in TME. Therefore, how to recruit M1 like macrophages to the core of the tumor is the key issue at present [169]. TLR agonists are currently being tested in clinical settings to repolarize M2 into M1 subtypes [170]. A recent study has shown that the expression of collagen XVIII (ColXVIII), a basement membrane component, is significantly upregulated in BC, and ColXVIII, as a regulator of epidermal growth factor receptor tyrosine kinase (ErbB) signaling, can interact with ErbB1 and ErbB2 (also known as EGFR and human epidermal growth factor receptor 2 [HER2]) and α 6-integrin forms complexes to promote BC cell proliferation, involving a cascade reaction of its N-terminus and MAPK/ERK1/2 and PI3K/AKT [171]. In addition, the CSF-1/CSFR-1 blocker inhibits the recruitment of TAMs into the tumor nest and has anti-tumor effects in clinical models [172]. Combining several current strategies targeting TAMs with ICIs for preclinical testing is expected to improve effectiveness in tumor treatment [173]. In a preclinical study of GBM, it was found that TAM2s can enhance the immune suppression of GBM and may lead to the failure of glioma immunotherapy. Knocking out the mouse EMP3 gene can reduce the production of CCL2 and TGF-β1 by GBM cells. Inhibiting the polarization and recruitment of TAM2s, while inhibiting tumor development in mouse glioma models, and generating survival advantages in mice. On the other hand, in EMP3 knockout mouse tumors, anti-PD1 therapy produced a greater anti-tumor response. In addition, it was observed that the number of CD4 + and CD8 + T cells in GBM tumors increased while reducing the expression of PD-L1 [174], providing a useful research basis for improving immune exclusion in tumors.

## Conclusions

As mentioned above, changes in ECM stiffness and polarization direction of TAMs intensively affect tumor immune exclusion and regulate T cell infiltration within the tumor nest. Therefore, a better understanding of the interaction and regulatory dynamics between ECM stiffness, polarization direction of TAMs, and immune cells can promote drug delivery and T cell infiltration in connective tissue proliferative tumors, and improve the efficacy of immunotherapy and patient survival. Furthermore, ECM stiffness and polarization direction of TAMs are also involved in various pathological processes of malignant tumor progression and play a key regulatory role. Future in-depth research can promote them to become reliable targets for tumor treatment, which is crucial for improving the treatment effectiveness and survival outcomes of tumor patients.

## Data Availability

Not applicable.

## Abbreviations

BC: Breast cancer
CAFs: Cancer-associated fibroblasts
CAR-Ms: Chimeric antigen receptor macrophages
CAR-Ts: Chimeric antigen receptor T cells
CCA: Cholangiocarcinoma
CRC: Colorectal cancer
CTLA4: Cytotoxic T-lymphocyte-associated protein 4
ECM: Extracellular matrix
EMT: Epithelial mesenchymal transition
EV: Extracellular vesicles
FGF: Fibroblast growth factor
GAG: Glycosaminoglycan
GBM: Glioblastoma multiforme
HCC: Hepatocellular carcinoma
HGG: High-grade glioblastoma
ICIs: Immune checkpoint inhibitors
LOX: Lysine oxidase
NK: Natural killer
NSCLC: Non-small cell lung cancer
OS: Overall survival
PDAC: Pancreatic ductal adenocarcinoma
PD-1: Programmed cell death protein 1
PD-L1: Programmed cell death-ligand 1
PDGF: Platelet-derived growth factor
PTT: Photothermal therapy
TAMs: Tumor-associated macrophages
TGF-β: Transforming growth factor-β
TIDE: Tumor immune dysfunction and exclusion
TILs: Tumor-infiltrating lymphocytes
TIME: Tumor immune microenvironment
TME: Tumor microenvironment
Treg: Regulatory T cells
